# Comparison of dogs and humans in visual scanning of social interaction

**DOI:** 10.1098/rsos.150341

**Published:** 2015-09-30

**Authors:** Heini Törnqvist, Sanni Somppi, Aija Koskela, Christina M. Krause, Outi Vainio, Miiamaaria V. Kujala

**Affiliations:** 1 Department of Equine and Small Animal Medicine, Faculty of Veterinary Medicine, Helsinki, Finland; 2 Cognitive Science, Faculty of Behavioural Sciences, University of Helsinki, Helsinki, Finland; 3 Neuroscience and Biomedical Engineering, Aalto University, Espoo, Finland

**Keywords:** domestic dog, eye movement, eye tracking, social interaction

## Abstract

Previous studies have demonstrated similarities in gazing behaviour of dogs and humans, but comparisons under similar conditions are rare, and little is known about dogs' visual attention to social scenes. Here, we recorded the eye gaze of dogs while they viewed images containing two humans or dogs either interacting socially or facing away: the results were compared with equivalent data measured from humans. Furthermore, we compared the gazing behaviour of two dog and two human populations with different social experiences: family and kennel dogs; dog experts and non-experts. Dogs' gazing behaviour was similar to humans: both species gazed longer at the actors in social interaction than in non-social images. However, humans gazed longer at the actors in dog than human social interaction images, whereas dogs gazed longer at the actors in human than dog social interaction images. Both species also made more saccades between actors in images representing non-conspecifics, which could indicate that processing social interaction of non-conspecifics may be more demanding. Dog experts and non-experts viewed the images very similarly. Kennel dogs viewed images less than family dogs, but otherwise their gazing behaviour did not differ, indicating that the basic processing of social stimuli remains similar regardless of social experiences.

## 1. Introduction

Domestic dogs (*Canis familiaris*) have become popular model animals in comparative cognition research, largely because of their excellent skills in comprehending human social communicative signals. Different theories of dogs' responsiveness to our social cues have been suggested: dogs learn to be responsive to human social cues during their interactions with humans [1], dogs have an innate gift for reading human social gestures selected for via domestication [2], dogs understand our mental states [3] or dogs are predisposed to learn about human communicative gestures [4].

Previous studies have shown that the gazing behaviour of dogs has similarities to that of humans. For example, when presented with a facial image, both species direct their attention to the socially informative facial features, e.g. the eye region [5,6], and demonstrate species-specific gaze bias towards the left hemiface [7]. A recent study also found similarities in the gazing behaviour of dogs and preverbal infants to human communicative signals [8]. However, comparisons of humans and dogs under similar conditions are rare, and little is known about the dogs' visual attention to natural, ecologically valid social scenes.

Human attention is drawn to the meaningful regions based on the viewer's knowledge [9], and similar top-down guidance of image gazing has also been found with eye-tracking studies of non-human primates [10–12]. Indications of the similar top-down guidance of gaze behaviour also exist in dogs. For example, dogs have been found to fixate more on familiar faces and eyes than on strange ones [6], and to discriminate between human facial expressions [13,14].

A recent study with naturalistic social interaction images also showed that monkeys and humans share a homologous social attention strategy when processing social scenes, and that the social attention seems to be modulated by experience [12]. Similarly, previous findings suggest that social experiences might also have an effect in the gazing behaviour of dogs [6].

Here, we examined the visuo-social gazing behaviour of dogs and humans to distinguish both similarities and differences between the two species. We recorded the eye movements of dogs while they viewed images containing humans or dogs either interacting with each other or facing away, and the results were compared with the equivalent data gathered from humans. Furthermore, we compared the gazing behaviour of two dog populations living in different social environments to clarify the effects of life experiences on the processing of social stimuli. Additionally, we compared the eye gaze patterns of two human groups, dog experts and non-experts, to see whether the expertise would affect the observation of the images.

We expected that, in accordance with previous studies [6,15], the social experiences of both humans and dogs have an effect on their gazing behaviour of social stimuli. We hypothesized that the family dogs gaze at the social stimuli more than the kennel dogs, and that the family dogs differentiate the social situations better than the kennel dogs.

## 2. Material and methods

### 2.1 Subjects

Altogether 46 dogs (38 privately owned family dogs, eight kennel dogs) and 26 humans (13 experts and 13 non-experts) participated in the study; the human data were a completely re-analysed subsample from a previous study with different goals [16].

Family dogs were 1–10 years old (5.1±2.0 years (mean±s.d.)), living in their owners' homes (31 females and seven males), representing 18 different breeds and three mongrels. They were fed once/twice a day and taken outdoors three to five times. Due to technical difficulties or restless behaviour of the dog, six family dogs (lacking eye-tracking data for more than 30% of the images) were excluded from further analyses.

Kennel dogs were eight 6-year-old healthy purpose-bred beagle dogs (two females and six males) living as a group in the kennel facilities of the University of Helsinki. They were fed twice a day and released into an outside exercise area once a day for 2 h. Kennel dogs regularly saw other beagle dogs living in the kennel, but they did not meet dogs of other breeds. In addition, they seldom met humans other than familiar caretakers and three researchers.

Dog experts (nine females, four males, age 31.9±6.6 years) were dog owners and had extensive experience of dogs, for example long-lasting involvement in hobbies such as agility or obedience training. Non-experts (five females, eight males, age 28.2±7.5 years) did not own a dog nor did they have extensive experience of dogs.

### 2.2 Training of the dogs

The dogs were pre-trained with positive operant conditioning method (clicker) to lie down and lean their head on a U-shaped chin rest during the presentation of the images, as previously [6,17]. Family dogs were trained by their owners and kennel dogs by the experimenters. Dogs were not encouraged to fix their eyes on a monitor or images during the training. The dogs were trained to perform the task on a voluntary basis without commands, and they were not physically restrained; for more details of the training, see Somppi *et al.* [17].

#### 2.2.1 Eye movement tracking and calibration

The dog measurements were conducted at the Faculty of Veterinary Medicine at the University of Helsinki. Binocular eye movements of the dogs were recorded with an infrared-based contact-free eye-tracker with a sampling rate of 250 Hz, based on a corneal reflection (iView X^^TM^^ RED250, SensoMotoric Instruments GmbH, Berlin, Germany), which was integrated into an LCD monitor. The monitor was placed at 0.51–0.75 m distance from the dog's eyes, depending on the size of the dog. The monitor, eye tracker and chin rest were in a cardboard cabin (*h*=1.5 m, *w*=0.9 m, *d*=0.9 m) with three walls and a roof. Two additional fluorescent lamps were placed in front and above the monitor. The illumination intensity measured on top of the chin rest was 4200–13 400 lx (11 000±2300 lx).

The eye tracker was calibrated for each dog's eyes using a five-point procedure [17]. The calibrated area was equal to a visual field of 40.5°×24.4° from the distance of 0.70 m. For calibration, the screen was replaced with a plywood wall with five holes in the calibration point positions in which the experimenter showed a treat to catch the dog's attention; for verification, two additional calibration check trials followed this initial calibration. To get an optimal calibration, 1–27 repeats (5±4) were required for each dog. The average calibration accuracy was 96% calculated as a proportion of fixated points out of five calibration points over two calibration checks of all dogs. Calibration and the experimental sessions were run on separate days to maintain ideal vigilance. The head position, illumination and position of the eye tracker were kept the same during the calibration, calibration checks and experimental sessions.

Human measurements were performed at the Advanced Magnetic Imaging Centre, Aalto University. Humans' eye movements were recorded with the SMI MEye Track long-range eye-tracking system with a sampling rate of 60 Hz, based on video-oculography and dark pupil-corneal reflection (SensoMotoric Instruments GmbH). The stimuli were viewed binocularly at a distance of 34 cm. Before the experiment, the eye tracker was calibrated using five fixation points; see Kujala *et al.* [16] for more details.

#### 2.2.2 Stimuli

**Figure 1.**
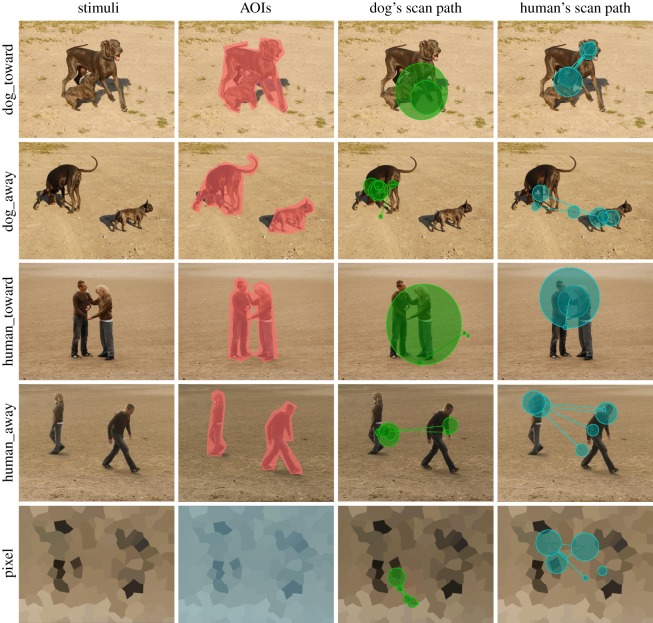
Left: examples of the stimuli. Middle left: the AOIs marked in different colours, object area in red and the whole image area in blue in the pixel stimulus. Middle right: an example of a dog's scan paths; and right: an example of a human's scan paths to the stimuli. The circles represent fixations and the lines trace the path that the eye travelled across the image.

The stimuli were taken from a previous study with humans [16]: a selection of 60/200 original stimuli were chosen for the dog study. The selected stimuli were colour photos of two dogs facing towards each other and greeting by sniffing or playing (*Dog_toward*); two dogs facing away (*Dog_away*); two humans facing each other and greeting, for example, by shaking hands or hugging (*Human_toward*); two humans facing away (*Human_away*); and crystallized pixel images, manufactured from a random sample of both interactive (*toward*) and non-interactive (*away*) image conditions (*Pixel*), as control stimuli (12 images per category, figure 1). The physical dimensions of the images were equal in dog and human studies (20×14 cm). In dogs, the pixel resolution of the images was 567×397 pixels, overlaid on a grey background of 1680×1050 px, and in humans, 640×480 pixels overlaid on a grey background of 1024×768 px.

#### 2.2.3 Experimental procedure

In the beginning of the experiment, the dog was released to the test room to settle down at the pre-trained position, while the experimenter and the dog's owner remained invisible to the dog behind an opaque barrier. The dog's behaviour was monitored through a webcam (Labtec Webcam 2200) placed on top of the monitor.

For dogs, the images were shown on a 22 inch (47.4×29.7 cm) LCD monitor using Experiment Center v. 3.0^^TM^^ software (SensoMotoric Instruments GmbH). The stimuli were shown during two separate days (30 images per day) in a pseudorandomized order. The stimuli were presented in four blocks of six to nine images, 2.5 s per stimulus. After every block, the dog was rewarded with a treat regardless of its gazing behaviour.

For humans, the images were shown on a projection screen by a data projector (Christie Vista x3, Christie Digital Systems Inc., USA), and the stimulus presentation was controlled with Presentation^®^ software (Neurobehavioral Systems, San Francisco, CA, USA). The stimuli were shown in two recording sessions in pseudorandomized order. Each image was shown for 2.5 s in a continuous 25-s stimulus block, 10 images per block, and the block alternated with 25-s rest blocks; for details, see Kujala *et al.* [16]. Before the experiment, the subjects were informed that they would see images of people, dogs and abstract pixel images. They were instructed to explore the images freely and inspect the attitude of the beings towards one another or towards their surroundings.

#### 2.2.4 Data processing

Successful eye gaze recordings were obtained from 32 family dogs and eight kennel dogs, on average 56±5 images per dog. In total, 147 images were excluded from the analysis due to eye-tracker software crash or dog leaving/lifting its head from the chin rest. In humans, on average 58±8 images per human were analysed. Successful eye gaze recordings for all of the 60 images were obtained from 24 of 26 participants; for two participants, 30 of the 60 images were excluded due to technical difficulties.

To render the dog and human data comparable, the data of both species were treated with the same procedures. Raw eye movement data were analysed using BeGaze v. 2.4^^TM^^ software (SensoMotoric Instruments GmbH). The fixation of the gaze was scored with a low-speed event detection algorithm that calculates potential fixations with a moving window spanning consecutive data points. The fixation was coded if the minimum fixation duration was 75 ms and the maximum dispersion value *D*=250 px (*D*=[max(*x*) − min(*x*)]+[max(*y*)−min (*y*)]). Otherwise, the recorded sample was defined as part of a saccade.

#### 2.2.5 Statistical analyses

Stimuli were divided into two areas of interest (AOI): (i) image area comprising the whole image but not the grey background, and (ii) object area in other than pixel images, comprising the heads and bodies of the two dogs/humans depicted in the images (figure 1). From the dogs' binocular raw data, total gaze time (sum of durations from all fixations and saccades that hit the AOI) was calculated for the image area and relative gaze time (the total gaze time targeted to the object area divided by the total gaze time targeted to the image area) for the object area. In addition, the number of saccades between two objects (the number of transitions of fixations from left object to right object and right object to left object) in the image was calculated. In humans, the total gaze time of the image area, relative gaze time of the object area and number of saccades between two objects in the images were calculated from monocular raw data for these two AOIs [18].

The statistical analyses were conducted using SPSS statistics v. 22.0 (IBM, New York, NY, USA). The total gaze times of family and kennel dogs in image area were compared with repeated-measures analysis of variance (ANOVA) with a between-subjects factor ‘*group*’ (family, kennel) and within-subjects factor ‘*category*’ (human_toward, human_away, dog_toward, dog_away, pixel). In addition, the total gaze times of family and kennel dogs for pixel images were compared with repeated-measures ANOVA with a between-subjects factor ‘*group*’ (family, kennel) and within-subjects factor ‘*behavior*’ (toward, away). The relative gaze times of family and kennel dogs were compared in a separate ANOVA with a between-subjects factor ‘*group*’ and within-subjects factors ‘*species*’ (human, dog) and ‘*behavior*’ (toward, away). Also, the number of saccades between two objects was inspected in ANOVA with a between-subjects factor ‘*group*’ and within-subjects factor ‘*category*’ (human_toward, human_away, dog_toward, dog_away). The ANOVA results were clarified with independent samples *t*-tests (between-groups comparisons) and paired-samples *t*-tests (within-groups comparisons). The gaze behaviour of experts and non-experts were compared with the same analysis as dogs' gaze behaviour, except with the between-subjects factor ‘*group*’ (expert, non-expert).

## 3. Results

### 3.1 Dog gaze behaviour

The total gazing time at the image area differed between family and kennel dogs (between-subjects factor *group*, *F*_1,38_=7.6, *p*<0.01, repeated-measures ANOVA). In addition, a main effect of *category* was found (*F*_4,152_=2.5, *p*<0.05). The planned comparisons with independent samples *t*-tests showed that family dogs gazed at images longer than kennel dogs in human_toward (1557±83 and 1058±119 ms, respectively; *t*_38_=2.8, *p*<0.01), human_away (1544±88 and 1056±128 ms, respectively; *t*_38_=2.6, *p*<0.05), dog_toward (1462±92 and 929±132 ms, respectively; *t*_38_=2.7, *p*<0.05) and dog_away (1460±79 and 930±96 ms, respectively; *t*_38_=3.2, *p*<0.01) categories, but no differences in gazing times were found in the pixel category (1441±96 and 1070±104 ms, respectively; *t*_38_=1.9, *p*=0.07). Within-groups comparisons revealed that family dogs gazed at the image area longer in human_toward than pixel (*t*_31_=2.5, *p*<0.05), human_away than pixel (*t*_31_=2.5, *p*<0.05) and human_away than dog_away categories (*t*_31_=2.1, *p*<0.05; paired-samples *t*-tests). In kennel dogs, the image area gazing time was longer in human_away than dog_away category (*t*_7_=2.4, *p*<0.05).

The total gazing time at toward and away pixel images did not differ between family and kennel dogs (between-subjects factor *group*, *F*_1,38_=3.1, *p*=0.08, repeated-measures ANOVA). The planned comparisons with paired-samples *t*-tests showed that across groups, there were no differences in the gazing times of toward and away pixel images (1358±94 and 1391±84 ms, respectively; *t*_39_=0.5, *p*=0.6).

**Figure 2.**
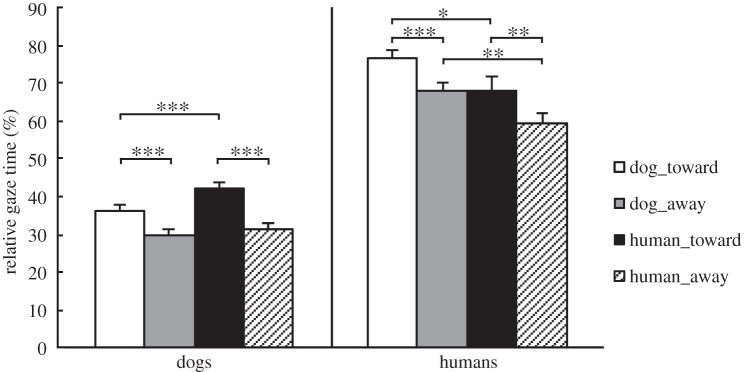
The differences between stimulus categories in the relative gaze time of the object area (+s.e.m.) by subject groups. The relative gaze time was calculated by dividing the total gaze time of the object area by the total gaze time of the image area. Statistically significant differences between the stimulus categories are represented by asterisks (^***^p<0.001, **p<0.01 and *p<0.05).

The relative gazing time at the object area did not differ between family and kennel dogs (between-subjects factor *group*, *F*_1,38_=0.6, *p*=0.5, repeated-measures ANOVA). Instead, main effects of *species* (*F*_1,38_=7.1, *p*<0.05) and *behavior* (*F*_1,38_=22.2, *p*<0.001) were found. The planned comparisons with paired-samples *t*-tests showed that both dog groups gazed relatively longer at the object area in interaction images and human images: gaze time was longer in human_toward than human_away (42±1.5 and 31±1.8%, respectively; *t*_39_=5.9, *p*<0.001), dog_toward than dog_away (36±1.5 and 30±1.4%, respectively; *t*_39_=4.9, *p*<0.001) and human_toward than dog_toward (42±1.5 and 36±1.5%, respectively; *t*_39_=13.3, *p*<0.001) images (figure 2).

The number of saccades between objects did not differ between family and kennel dogs (between-subjects factor *group*, *F*_1,22_=4.1, *p*=0.06, repeated-measures ANOVA). However, a main effect of *category* was found (*F*_3,66_=9.1, *p*<0.001). The planned comparisons with paired-samples *t*-tests showed that across groups, dogs exhibited more saccades between objects in human_toward than in human_away images (0.2±0.03 and 0.03±0.01, respectively; *t*_23_=4.9, *p*<0.001).

### 3.2 Human gaze behaviour

The total gazing time at the image area did not differ between experts and non-experts (between-subjects factor *group*, *F*_1,24_=0.4, *p*=0.5, repeated-measures ANOVA). However, a main effect of *category* was found (*F*_2,47_=6.0, *p*<0.01). The paired-samples *t*-tests showed that in both groups, pixel images were gazed at longer than human_away images (2313±55 and 2110±91 ms, respectively; *t*_25_=3.4, *p*<0.01) and dog_away images were gazed at longer than human_away images (2284±67 and 2110±91 ms, respectively; *t*_25_=3.5, *p*<0.01).

The total gazing time at toward and away pixel images did not differ between experts and non-experts (between-subjects factor *group*, *F*_1,24_=0.4, *p*=0.5, repeated-measures ANOVA). The paired-samples *t*-tests revealed that across groups, there were no differences in the gazing times of toward and away pixel images (2341±61 and 2307±56 ms, respectively; *t*_25_=1.0, *p*=0.3).

The relative gaze time at the object area did not differ between experts and non-experts (between-subjects factor *group*, *F*_1,24_=0.5, *p*=0.5, repeated-measures ANOVA). Instead, main effects of *species* (*F*_1,24_=12.3, *p*<0.01) and *behavior* (*F*_1,24_=40.3, *p*<0.001) were found. The planned comparisons with paired-samples *t*-tests showed that across groups, object area was gazed relatively longer in human_toward than human_away (68±3.7 and 59±2.9%, respectively; *t*_25_=3.6, *p*<0.01), dog_toward than dog_away (77±2.5 and 68±2.3%, respectively; *t*_25_=4.9, *p*<0.001), dog_toward than human_toward (77±2.5 and 68±3.7%, respectively; *t*_25_=2.6, *p*<0.05) and dog_away than human_away images (68±2.3 and 59±2.9%, respectively; *t*_25_=3.4, *p*<0.01; figure 2).

The number of saccades between objects did not differ between experts and non-experts (between-subjects factor *group*, *F*_1,22_=0.001, *p*=0.9, repeated-measures ANOVA). However, a main effect of *category* was found (*F*_2,42_=6.2, *p*<0.01). The planned comparisons with paired-samples *t*-tests showed that across groups, humans exhibited more saccades between objects in dog_toward than human_toward images (1.4±0.07 and 0.9±0.13, respectively; *t*_23_=3.6, *p*<0.01), and in dog_away than human_away images (1.4±0.07 and 1.1±0.05, respectively; *t*_23_=3.1, *p*<0.01).

## 4. Discussion

Here, we exhibit the first evidence of the similarities and dissimilarities of humans and dogs in the visual scanning of natural social scenes containing gestural communication between conspecifics or non-conspecifics. Comparing the results between dogs and humans, we found that both dogs and humans gazed longer at the object area in social interaction images than non-social images. The category-related differences in the object area suggest that dogs were able to differentiate social and non-social stimuli from each other, and that they preferred the social stimuli, as previously observed in human infants [19]. The gazes of the actors in the social interaction images can guide the subjects' viewing from one head to another, whereas in the non-social images the subjects may spend more time following the gazes pointing elsewhere [16]. Infants and adult humans attend automatically to where someone else is looking [20], and animals also appear to share this basic gaze-following behaviour [21]. Since the gazing times between interactive and non-interactive pixel images did not differ in either humans or dogs, the observed difference between actors in the social interaction images and non-social images is unlikely to be explained only by their responses to the low-level stimulus properties such as the distance between actors in the images [9,22].

Dogs and humans also exhibited differences in their gazing behaviour. Dogs gazed longer at the object area in human than dog social interaction images, whereas humans gazed longer at the object area in dog than human social interaction images. In addition, dogs made more saccades between objects in human social interaction than in human non-social images, suggesting that human interaction images required closer scanning of body postures than non-social images. Instead, humans exhibited more saccades between objects in dog than in human social interaction images, and in dog than in human non-social images. Thus, as both species gazed longer at objects in non-conspecific images and also made more transitions between objects in the non-conspecific images, this could indicate that processing social interaction of non-conspecifics may be more demanding and require more detailed analysis. Previous research with humans has shown that saccadic eye movements can be modulated by the cognitive demand and characteristics of the observed scene [23,24].

Dogs' gazing behaviour might also reflect their excellent skills in comprehending human communicative cues [1,25] that are abundantly present in the human social interaction images. The ability to respond appropriately to human cues is argued to have been a selective advantage during domestication for dogs [26,27], thus it is not surprising that dogs are also interested in the human interactive cues in images (e.g. gazing or leaning towards each other). In our previous studies using only faces instead of whole-body natural scenes, dogs have preferred conspecific faces to human faces [6,17]. This difference could be due to the more complex social images presented here, featuring both faces and bodies as opposed to purely facial images presented in earlier studies (see also, e.g. [12]).

Between the different dog groups, family dogs gazed at the image area longer than the kennel dogs in all other stimulus categories but pixel images, where their gazing times did not differ. Although a clear interaction effect between dog group and stimulus category was not found, the results of the planned *t*-tests suggest that kennel dogs' limited social environment and life experiences might have had an effect on the processing of the natural social stimuli. Family dogs might be more interested in the natural images, because they have had more opportunities for social interactions with dogs and humans than have kennel dogs. In addition to the social background, the dog's breed can influence the gazing behaviour [28–30]. Here, the kennel dogs were hunting dogs, whereas most of the family dogs were herding or working dogs, which might affect their motivation and ability to search and interpret human communicative cues.

The relative gaze time at the object area and the number of saccades between objects did not differ between family and kennel dogs. Contrary to our expectations, it would seem that family and kennel dogs are able to differentiate the object-level details in the social situations similarly. Both family and kennel dogs attended to the meaningful and informative areas in the images, which indicates that basic processing of images is similar across dogs regardless of the living environment. These results are in line with our earlier study with facial images. We found that family dogs fixated on facial images longer than kennel dogs, but they responded to inversion, familiarity and species conditions similarly, which are associated with basic mechanisms of face processing [6].

The gazing time at the image area, relative gaze time at the object area and number of saccades between objects did not differ between experts and non-experts in this study. The relatively small sample size might have affected the results. Nevertheless, this is in accordance with previous study, where the total fixation durations did not differ between experts and non-experts. However, in the previous study dog experts gazed relatively longer at dog bodies than dog heads compared to the non-experts [16], suggesting a small effect of expertise remaining in examining dog body postures.

In any eye-tracking study, stimulus characteristics play a central role in image viewing patterns. The ecological validity has an impact on the results, reflected in the differences between gazing at isolated faces, scenes and videos [31,32]. Here, the images depicted real-life social interactions, but videos from social situations might be even more naturalistic [33], maybe contributing to the observation of conspecifics by dogs. Nevertheless, video brings a fourth dimension to the analysis rendering the stimuli more uncontrollable, and in this study we wanted to lay a more controlled basis for the study. Also using a head-mounted eye tracker would allow the examination of eye movements of subjects who are freely moving and interacting in real-life environments [34–36], but a cross-species analysis of this kind of recordings might be problematic.

In conclusion, both dogs and humans gazed longer at objects in social interaction images than those in non-social images. Nonetheless, they also exhibited differences in their gazing behaviour. Both species gazed longer at objects in non-conspecific images and made more saccades between objects in images representing non-conspecifics, which could indicate that processing social interaction of non-conspecifics may be more difficult and require more detailed analysis. Dogs' interest towards human social images might also reflect their skills in comprehending human communicative cues. Family dogs were generally more interested in the images than kennel dogs, but the basic processing of the social stimuli would appear to be similar across dogs regardless of their social experiences.

## Data accessibility

Data are available from the Dryad Digital Repository: http://dx.doi.org/10.5061/dryad.9rn92.
